# Supplementary material for: Deletion of wfs1 Impairs Oligodendrocyte Precursor Cells Dorsal Distribution and Myelination Through the wfs1-hmgcs1 Axis in Zebrafish
Source: Biology (Basel). 2026 Aug 16;15(16):1402. doi: 10.3390/biology15161402 (PMC13510151; doi:10.3390/biology15161402)
Supplement: Supplementary file 1 [file biology-15-01402-s001.zip › File S1-Transcriptome sequencing data and differentially expressed genes (DEGs) in wfs1-- zebrafish at 5 dpf/2.KEGG/wfs1KOvsWT/DOWN/src/dre04261.html]

dre04261


- dre:559015

- Down regulated genes

559015(-5.59690131475721)
- dre:678560

- Down regulated genes

678560\_1(-3.312043389824)

- dre:353247

- Down regulated genes

353247(-1.16019504230133)

- dre:100037358

- Down regulated genes

100037358\_1(-1.14225112918381)

- dre:64614

- Down regulated genes

64614(-1.08866039335654)
- dre:114457

- Down regulated genes

114457(-1.18395641076668)

- dre:100333318

- Down regulated genes

100333318(-1.02298964704357)

- dre:555423

- Down regulated genes

555423(-1.09041761243408)

- dre:562282

- Down regulated genes

562282\_1(-5.61712483666314)
- dre:559392

- Down regulated genes

559392(-2.91317811245432)

- dre:497384

- Down regulated genes

497384\_1(-2.05267237809407)

- dre:562282

- Down regulated genes

562282\_1(-5.61712483666314)
- dre:559392

- Down regulated genes

559392(-2.91317811245432)

- dre:492486

- Down regulated genes

492486\_1(-1.65362606223537)

- dre:562282

- Down regulated genes

562282\_1(-5.61712483666314)
- dre:559392

- Down regulated genes

559392(-2.91317811245432)

- dre:550357

- Down regulated genes

550357\_2(-4.61593897492172)

- dre:793666

- Down regulated genes

793666(-1.15928527641875)

Close
